# Diagnosis, Treatment, and Outcome in Patients with Bleeding Peptic Ulcers and *Helicobacter pylori* Infections

**DOI:** 10.1155/2014/658108

**Published:** 2014-06-30

**Authors:** Ting-Chun Huang, Chia-Long Lee

**Affiliations:** ^1^Division of Gastroenterology, Department of Internal Medicine, Cathay General Hospital, Taipei 10650, Taiwan; ^2^School of Medicine, Fu-Jen Catholic University, New Taipei City 24205, Taiwan; ^3^Division of Gastroenterology, Department of Internal Medicine, Hsinchu Cathay General Hospital, No. 678, Section 2, Junghua Road, Hsinchu 30060, Taiwan; ^4^School of Medicine, College of Medicine, Taipei Medical University, Taipei 11031, Taiwan

## Abstract

Upper gastrointestinal (UGI) bleeding is the most frequently encountered complication of peptic ulcer disease. *Helicobacter pylori* (*Hp*) infection and nonsteroidal anti-inflammatory drug (NSAID) administration are two independent risk factors for UGI bleeding. Therefore, testing for and diagnosing *Hp* infection are essential for every patient with UGI hemorrhage. The presence of the infection is usually underestimated in cases of bleeding peptic ulcers. A rapid urease test (RUT), with or without histology, is usually the first test performed during endoscopy. If the initial diagnostic test is negative, a delayed ^13^C-urea breath test (UBT) or serology should be performed. Once an infection is diagnosed, antibiotic treatment is advocated. Sufficient evidence supports the concept that *Hp* infection eradication can heal the ulcer and reduce the likelihood of rebleeding. With increased awareness of the effects of *Hp* infection, the etiologies of bleeding peptic ulcers have shifted to NSAID use, old age, and disease comorbidity.

## 1. Introduction

Left untreated, peptic ulcer diseases (PUD) will cause major complications, such as hemorrhage, perforation, or obstruction in 20–25% of patients. Among these complications, upper gastrointestinal (UGI) bleeding is the most frequently encountered, accounting for about 70% of cases [1, 2]. With the discovery of* Helicobacter pylori* (*Hp*) [3], the pathogenic relationship between PUD and* Hp* infection has come into focus. Worldwide consensus guidelines recommend the mandatory eradication of* Hp* in patient with PUD [4–13].

Another independent risk factor for PUD and subsequent UGI bleeding is the administration of nonsteroidal anti-inflammatory drugs (NSAIDs) [14]. Those patients requiring long-term NSAID treatment should be screened for* Hp* status, and* Hp* eradication is suggested before administering NSAIDs [8]. Writing prescriptions for aspirin and antiplatelet agents is a common clinical scenario that creates new challenges related to UGI bleeding in gastroenterological practices [15, 16]. However, the relationship between the use of these medications and UGI bleeding is beyond the scope of this paper. Here, we will elucidate the relationship between bleeding peptic ulcers and* Hp* infection from the chronological perspective with an emphasis on diagnosis, treatments, and outcomes.

## 2. Materials and Methods

We searched Pubmed (to 15 March 2014). Overall, we identified 708, 526, and 120 with the following key word combinations: “bleeding peptic ulcer AND* Helicobacter pylori* diagnosis,” “bleeding peptic ulcer AND* Helicobacter pylori* treatment,” and “bleeding peptic ulcer AND* Helicobacter pylori* outcome,” respectively.

Medical subject headings (MeSH) terms were employed to assist the search, and the results were reviewed by the authors. We also conducted a manual search of material from several congresses. The paper selection criteria included (1) discussion with diagnosis, treatment, or outcome of bleeding peptic ulcers and* Hp* infection and (2) publication in full manuscript form in English. Finally, 129 articles were selected, and their reference lists were checked for other possible studies for inclusion.

## 3. Results and Discussion

### 3.1. Diagnosis

The diagnosis of* Hp* infection is based on both invasive and noninvasive methods. Endoscopy is an invasive method, which includes a rapid urease test (RUT), histology, culturing, and polymerase chain reaction (PCR). The noninvasive methods include serology antibody assessment, ^13^C-urea breath test (UBT), and stool antigen testing. There are only minimal differences in the accuracies of the invasive tests. Among them, the RUT is the most frequently used. The UBT is the recommended noninvasive test [17]. Recently, the monoclonal stool antigen test has also been suggested [18]. The prevalence of* Hp* infection in noncomplicated PUD has been reported to be high in duodenal ulcer patients and moderate in gastric ulcer patients, regardless of which test is performed [19, 20]. However, there have been discrepant test results among patients with bleeding peptic ulcers. The individual diagnostic tests are discussed below.

#### 3.1.1. RUT

The RUT is the most common examination for patients with UGI bleeding because endoscopy is always performed in such cases. An early study from Hong Kong disclosed a high false-negative rate for urease tests from antral biopsies in bleeding ulcer patients [21]. Almost simultaneously, we reported delayed positive results on the CLO test (color change after 24 hours) in our bleeding peptic ulcer patients if there was blood in the gastric antrum [22]. Another study from Greece demonstrated similar results at the same meeting [23]. These studies were further elucidated in subsequently published full articles [24–26].

Because there is always blood in the stomachs of patients with bleeding peptic ulcers, interference with RUT results by blood components is a concern. Several mechanisms have been suggested, including the bactericidal effect of serum inducing a transient decrease in bacterial density, the presence of anti-*Hp* antibodies inhibiting urease production, suppressed urease activity by serum enzymes or electrolytes, various buffering systems (e.g., albumin, bicarbonate, and phosphate) interfering with the pH level of the RUT reagent, and concomitant administration of NSAIDs or proton pump inhibitors (PPIs). In one in vitro study [27], a false-negative RUT result was caused by the buffering effects of serum albumin on the pH indicator but not on urease activity. Another in vitro study concluded that large gastric lavage before endoscopy can cause a false-negative RUT result [28]. However, our study found no influence on the likelihood of a false-negative result if the gastric antral biopsy specimen was cleansed by normal saline before inoculating the wells for the CLO test [29]. Similarly, another study concluded that an artificial blood-soaked antral specimen did not influence the results of two RUTs [30]. The bactericidal effect of human plasma [31, 32] and the reduction in bacterial load by PPIs [33] have been demonstrated.

In subsequent studies worldwide [34–40], RUT was further confirmed to be less sensitive than other tests in the diagnosis of* Hp* infection in bleeding peptic ulcers. Another consideration is that* Hp* bacterial density may be patchy, and only using samples from the gastric antrum may be inadequate. Inappropriate biopsy site and inadequate specimens are other explanations of false-negative results of RUT in patients with UGI bleeding. (Blood in stomach could induce* Hp* migration to corpus and fundus and the decrease of bacterial density in the antrum. Fewer amounts of specimens are obtained during emergent endoscopy procedure.) Simultaneous antral and body specimens or multiple biopsies have been found to produce more positive RUTs [41, 42]. Most authors concluded that the RUT cannot be the only diagnostic test in such circumstances [43]. If the initial diagnostic test is negative, a delayed test 4–8 weeks later can have up to an 80% positive rate in previously negative patients [44].

#### 3.1.2. Histology

Different studies have reported low sensitivity with histologic methods, which is consistent with RUT sensitivity. This suggests that histology cannot reliably exclude* Hp* infection in patients with bleeding peptic ulcers [24, 34]. However, other studies have reported that histology is more sensitive than RUT [24, 25, 35, 36]. As previously mentioned, patchy distribution of bacterial density can be one factor, but the staining method and pathologist's interpretations also influence the results [45]. Others have suggested that the prevalence of* Hp* infection is probably the same among bleeding and nonbleeding patients [46]. The sensitivity of histology also relies on the experience of the endoscopist to take the biopsy from the appropriate site. Some publications had shown that atrophic change, rugal hyperplasia, edema, and spotty erythema are valuable endoscopic findings of* Hp* infection. It is very important to avoid false-negative histology finding by taking the biopsy from RAC (regular arrangement of collecting venules) negative site [47]. Therefore, combination tests should be performed to achieve a more precise diagnosis [48].

#### 3.1.3. Culturing and PCR

Culturing* Hp* in patients with bleeding peptic ulcers produced a low yield in several studies [24, 34]. The reasons for its infrequent use include the time-consuming nature of the process due to the microanaerobic pathogen characteristics and the lack of time to perform the procedure during endoscopy.

Mucosal PCR has been used as an invasive test to diagnose* Hp* infection. In one study, this test was less sensitive in patients with bleeding peptic ulcers than for those with nonbleeding peptic ulcers and chronic gastritis [49]. However, another study reported that PCR had higher sensitivity than other biopsy-based tests and similar sensitivity to noninvasive tests [50]. The authors also demonstrated that blood may reduce the sensitivities of all biopsy-based tests. A study using real-time PCR can improve* Hp* detection in a histology-negative, formalin-fixed, and paraffin-embedded biopsy and is superior to immunohistochemical staining [51]. Modified PCR could improve diagnostic accuracy in patients with UGI bleeding [52].

#### 3.1.4. UBT

Many studies have confirmed that the ^13^C-UBT can accurately diagnose* Hp* infection [53, 54]. This statement also applies to patients with UGI bleeding [24, 26, 35, 36, 38]. The test's sensitivity is not affected by blood in the stomach and is higher than those of biopsy-based methods and other noninvasive tests [55–57].

Because a subject must drink a urea-containing solution in conjunction with a test meal or citric acid, one might question whether this method is suitable for bleeding patients. Most UBTs are done when patients resume eating, or the UBT is reserved as a delayed test if the initial invasive methods are negative. However, using low-dose encapsulated ^13^C-urea has proven to be feasible in fasting patients or even before an endoscopy because it only takes a small amount of water to swallow a pill [58].

#### 3.1.5. Stool Antigen Test

The stool* Hp* antigen test has been introduced as an accurate noninvasive test [59]. It can be performed by enzyme-linked immunosorbent assay (ELISA) with monoclonal or polyclonal antibodies or by immunochromatographic assay with monoclonal antibodies. The sensitivity of this method is reduced by UGI bleeding when polyclonal ELISA or immunochromatographic stool antigens are used [38, 60, 61]. Furthermore, it is not reliable in patients with bleeding peptic ulcers [62]. Another study reported a high number of false-positive results in patients with UGI bleeding due to a cross-reaction with the blood [63]. Therefore, the stool* Hp* antigen diagnostic test is not recommended for use in patients with UGI.

#### 3.1.6. Serology

We [24] and others [34] have demonstrated that serology is more sensitive than other invasive tests in cases of bleeding peptic ulcer. It can be used as the initial invasive test, as an alternative test, or when the UBT test is negative. However, commercial serological tests must be confirmed by a local laboratory before they are used in an individual hospital [64]. Additionally, if patients have been treated for* Hp* infection, serological tests have revealed that serum antibodies may last for up to a year [65]. This fact must not be overlooked when interpreting the results.

#### 3.1.7. NSAIDs, PPI, and Other Drugs with Bleeding PUD on Diagnosis Tests

No matter which diagnostic tests are employed in patients with bleeding PUD, physicians should preclude NSAID use. Many studies have confirmed the influence of NSAIDs on the sensitivities of the test results [66–68].* Hp* infection and NSAID use are two independent factors related to bleeding peptic ulcers [69]. In patients who are already on long-term NSAIDs,* Hp* eradication does not prevent the peptic ulcer from bleeding. Nevertheless, patients who require long-term NSAID medications should be tested for* Hp* infection in advance.* Hp* eradication can decrease the incidence of peptic ulcer bleeding. But in patients with long-term NSAIDs use, the cause of peptic ulcer bleeding should be NSAIDs use, not* H. pylori* status.

Another frequently encountered scenario is that most patients are given PPIs either intravenously or orally at the initial presentation of UGI bleeding, even before an endoscopic examination. There is concern over whether the recent use of a PPI interferes with the diagnostic accuracy for* Hp* infection. One study with a 3-day dosage of intravenous PPI in a bleeding peptic ulcer case found that a high infusion dose significantly impacts negative histology and RUT results as compared to a regular daily dose [70]. Dose-dependent PPIs do produce short-term effects on* Hp* diagnosis. Recent PPI use may induce false-negative results on both invasive tests [35] and noninvasive tests, such as the UBT [71–74] and stool* Hp* antigen test [75]. The duration of PPI administration can variably affect diagnostic accuracy. Usually, discontinuation of the drug for 2 weeks is recommended before performing any test, except serology.

Antisecretory medication is mandatory in patients with bleeding peptic ulcers. H2-receptor antagonists (H2RA) may be an alternative regimen. There are several studies evaluating H2RA and* Hp* diagnostic accuracy. Conflicting results exist, but most data indicate that these drugs have little influence on the* Hp* diagnosis [76, 77].

#### 3.1.8. Summary

A systematic review and meta-analysis explored the accuracies of* Hp* diagnostic tests in patients with bleeding peptic ulcers [78]. The authors found that biopsy-based methods had low sensitivity and high specificity; UBT had high accuracy; stool antigen tests were less accurate; and serology, though not influenced by UGI bleeding, was not recommended as the first test. Pooled data on sensitivity, specificity, and positive and negative likelihood ratios are shown in Table 1. Because the positive likelihood ratio is high, positive invasive tests or UBT requires no further confirmation of* Hp* infection. However, the other delayed tests should not be overlooked.

A recent meta-regression study [79] suggested that the low prevalence of* Hp* infection in patients with bleeding peptic ulcers might be related to the methodology of the studies and to the patients' characteristics. The authors found a higher prevalence of* Hp* infection when a delayed test was performed and when younger patients were included. They concluded that the prevalence of* Hp* infection had been underestimated in patients with bleeding peptic ulcers. They also suggested that a delayed diagnostic test should be carried out if the initial diagnostic test is negative, as recommended by the International Consensus [11].

### 3.2. Treatment

#### 3.2.1. *Hp *Eradication

*Hp *infection is still an important factor in peptic ulcer development. Eradication therapy is suggested for both duodenal and gastric ulcers in patients infected with* Hp* [13], regardless of whether they have complications. Although there is no direct causal relationship between* Hp* infection and early rebleeding in patients with peptic ulcer bleeding [80, 81], empirical* Hp* eradication as soon as patients resume eating is the most cost-effective strategy for preventing recurrent hemorrhage [82].

Many studies in the 1990s demonstrated the benefit of* Hp* eradication in decreasing peptic ulcer recurrences, as well as in bleeding cases. Using antibiotics to kill the bacteria was proven effective in preventing ulcer rebleeding in early studies [83, 84]. Other regimens using omeprazole and amoxicillin can also reduce the recurrence of peptic ulcer bleeding as compared to omeprazole or ranitidine alone [85–88]. The results were the same when the antibiotics were changed [89].

With the introduction of the ideal eradication regimen for* Hp* infection, triple therapy has been applied worldwide [90–92]. We previously reported that triple therapy can achieve a 91.3% eradication rate and a 97.1% ulcer healing rate in bleeding peptic ulcers [93]. One study found that as long as antibiotic eradication or* Hp* infection suppression is achieved, bleeding can be reduced [94]. Subsequent studies also confirmed that* Hp* eradication improves healing and decreases rebleeding [95–97].

The current dogma is that* Hp* eradication in bleeding peptic ulcers is superior to simple ulcer healing in preventing further ulcer hemorrhages [98, 99]. Therefore, testing for the presence of* Hp* infection and eradicating it are both mandatory and cost effective [100]. While there has been concern over whether maintenance antisecretory treatment was necessary, the current position is that, as long as* Hp* is eradicated, peptic ulcer rebleeding is virtually eliminated. Therefore, antisecretory therapy is no longer required [101–104]. However, maintenance antisecretory therapy should be considered for* Hp*-eradicated patients who did not stop NSAID use.

We performed a prospective 5-year followup of patients after* Hp* eradication and assessed the healing of bleeding peptic ulcers [101]. We randomized 82 consecutive patients into 4 different groups after 1 week of triple therapy and 3 weeks of PPI treatment. Despite 4 months of different maintenance regimens among the four groups (antacid suspension, colloidal bismuth, famotidine, or a placebo treatment), all the patients remained ulcer free with no evidence of reinfection. In recent pooled data of 1000 patients from 10 Spanish university hospitals and a total of 3253 patient-years of long-term followup, maintenance antiulcer treatment was not indicated once* Hp* had been eradicated [104]. However, the recent Maastricht IV/Florence Consensus suggested that while maintenance antiulcer treatment is not needed for bleeding duodenal ulcers, it should be continued for gastric ulcers [13].

PPI treatment is usually administered to patients with bleeding peptic ulcers, even before endoscopic examination. This treatment can facilitate the endoscopic hemostatic effect in reducing short-term rebleeding [105, 106]. PPI treatment also has benefits for* Hp* eradication. One study demonstrated that intravenous omeprazole can decrease the risk of peptic ulcer rebleeding and may even improve the* Hp* eradication rate of the subsequent triple therapy [107].

*Hp* eradication after peptic ulcer bleeding reduces recurrence. Is confirmation of eradication of* Hp* worthwhile? One study using the Markov model proved that confirmation of* Hp* eradication after completion of antibiotic treatment in peptic ulcer bleeding is cost effective [108].

#### 3.2.2. Summary

Eradication therapy is suggested in* Hp*-infected bleeding peptic ulcers. Triple therapy including a PPI and two antibiotics is the primary regimen. However, the rising antibiotic resistance rate should be taken into consideration in specific regions. Concomitant triple therapy, sequential therapy, bismuth- or non-bismuth-based quadruple therapy, and levofloxacin-based regimens are appropriate alternatives. After eradication, prolonged acid-suppressive therapy for duodenal ulcers is unnecessary, but gastric ulcers may require additional acid-suppressive therapy for 4–8 weeks due to their slow healing time and larger size.

### 3.3. Outcome

#### 3.3.1. Outcome with/without* Hp *Eradication

Among patients with peptic ulcer diseases, 20–25% develop bleeding, perforation, or obstruction. In patients with bleeding peptic ulcers, approximately 33% develop recurrent bleeding within 1-2 years if left untreated after the ulcer heals [117]. Consequently,* Hp* eradication reduces the recurrence rate of peptic ulcers [118]. As mentioned previously, several studies have also reported a low rebleeding rate after* Hp* eradication, even without acid-suppressive drug maintenance [83–89, 95, 96, 101, 109, 111–116]. A multicenter Spanish cohort study with similar findings was published recently, and comparative results with other studies are shown in Table 2.

Posttreatment* Hp* status has been found to be an independent predictor of duodenal ulcer bleeding recurrence [113]. Followup* Hp* testing after eradication in cases of bleeding peptic ulcers is therefore beneficial [108]. Because recrudescence is more common than reinfection [119], physicians should use combined tests or select a much lower cut-off value for ^13^C-UBT to verify eradication success.

Is there a trend toward decreasing* Hp*-related bleeding peptic ulcers today? The answer is yes. After the global implementation of* Hp* eradication for PUDs, the incidence of* Hp*-infected UGI hemorrhage has decreased. A 10-year nationwide database from Taiwan also demonstrated 42–48% and 41–71% decreases in the incidence of hospitalization for gastric ulcers and duodenal ulcers, respectively, and these rates included uncomplicated and complicated cases [120]. Similar results have also been reported in other countries [121].

Nevertheless, bleeding peptic ulcers remain a worldwide problem. The increasing use of NSAIDs is considered to be an important underlying cause. Many studies have confirmed that current UGI bleeding in patients can be attributed to NSAID usage [122–126]. One study from the United States found that admission for PUD-related complications has not decreased despite decreasing* Hp* prevalence and increasing* Hp* eradication [127], and the authors proposed that this could be due to NSAID use. Meanwhile,* Hp* eradication can decrease the long-term incidence of recurrent ulcer bleeding in low-dose aspirin users [128]. Eliminating one independent risk factor can attenuate the effect of another independent factor on inducing peptic ulcer bleeding. A recent study found that patients with bleeding peptic ulcers and concurrent* Hp* infection have a more favorable outcome than those without [129].

#### 3.3.2. Summary

*Hp* infection is an independent risk factor for bleeding duodenal ulcers.* Hp*-infected gastric ulcers in combination with old age and NSAID or aspirin therapy may increase the bleeding risk. Eradication treatment can decrease the likelihood of peptic ulcer rebleeding and associated complications. Admissions for bleeding peptic ulcers have not decreased despite the eradication of* Hp* infections. Concomitant administration of NSAIDs, old age, and comorbidities are currently considered as risk factors for UGI bleeding.

## 4. Conclusions

Three decades after the discovery of* Hp*, the etiologies of bleeding peptic ulcers are changing. However, diagnosis of* Hp* infection is still the first priority in these patients. Invasive RUT is most frequently used, but this methodology is hampered by a high rate of false-negative results, especially in patients with UGI bleeding. Other delayed tests should be performed if the initial diagnostic test is negative. Eradication of* Hp* infection can reduce the risk of rebleeding and should be started as soon as patients resume eating. Concomitant use of NSAIDs, aspirin, or other antiplatelet drugs associated with old age and comorbidities is the most likely etiology for current bleeding peptic ulcers.* Hp* eradication is beneficial for patients who require the long-term administration of these drugs.

## Figures and Tables

**Table 1:** Accuracies of different diagnostic tests based on pooled data of different studies of patients with bleeding peptic ulcers [78].

Diagnostic test	Number of studies	Pooled patients	Sensitivity	Specificity	Positive LR	Negative LR
RUT	16	1,417	0.67	0.93	9.6	0.31
Histology	10	827	0.70	0.90	6.7	0.23
Culture	3	314	0.45	0.98	19.6	0.31
UBT	8	520	0.93	0.92	9.5	0.11
Stool Ag	6	377	0.87	0.70	2.3	0.2
Serology	9	803	0.88	0.69	2.5	0.25

Ag: antigen; LR: likelihood ratio; RUT: rapid urease test; UBT: urea breath test.

**Table 2:** Incidence of rebleeding in *Hp*-eradicated patients with no maintenance acid-suppressive therapy among different studies [104].

Author	Year and area	Ulcer type	Regimen	ER number	Mean F/U (M)	Rebleeding number (%)
Graham et al. [83]	1993, USA	PU	Triple	17	12	0 (0%)
Labenz and Borsch [84]	1994, Germany	PU	7 different protocols	42	17	0 (0%)
Jaspersen et al. [86]	1995, Germany	PU	Dual	24	12	0 (0%)
Jaspersen et al. [109]	1995, Germany	DU	Dual	29	12	1 (3.4%)
Rokkas et al. [85]	1995, Greece	DU	Dual	13	12	0 (0%)
Santander et al. [87]	1996, Spain	PU	Dual or triple	84	12	2 (2.3%)
Riemann et al. [88]	1997, Germany	PU	Dual	42	19	2 (4.8%)
Sung et al. [89]	1997, Hong Kong	PU	Triple	108	12	0 (0%)
Macri et al. [110]	1998, Italy	DU	Quadruple	21	48	0 (0%)
Amendola et al. [111]	1999, Argentina	PU	PPI 1-week regimen	42	24	0 (0%)
Gisbert et al. [112]	1999, Spain	DU	Triple or dual	111	12	0 (0%)
Lai et al. [113]	2000, Hong Kong	DU	Triple	41	53	2 (4.9%)
Vergara et al. [95]	2000, Spain	PU	Triple or quadruple	93	27	0 (0%)
Pellicano et al. [114]	2001, Italy	DU	Antibiotics	46	47	0 (0%)
Capurso et al. [115]	2001, Italy	DU	Dual or triple	83	36	3 (3.3%)
Arkkila et al. [96]	2003, Finland	PU	Quadruple or dual	176	12	2 (1.1%)
Liu et al. [101]	2003, Taiwan	PU	Triple	26	56	0 (0%)
Horvat et al. [116]	2005, Croatia	GU	Triple	43	12	1 (2.3%)
Gisbert et al. [104]	2012, Spain	PU	Triple∗	1000	39	5 (0.5%)

DU: duodenal ulcer; ER: eradication; F/U: followup; GU: gastric ulcer; PU: peptic ulcer (gastric or duodenal ulcer).

∗Triple therapy first followed by 2nd-, 3rd-, or 4th-line treatment.
